# Genome-Wide Copy Number Variation Association Study of Atrial Fibrillation Related Thromboembolic Stroke

**DOI:** 10.3390/jcm8030332

**Published:** 2019-03-09

**Authors:** Chia-Shan Hsieh, Pang-Shuo Huang, Sheng-Nan Chang, Cho-Kai Wu, Juey-Jen Hwang, Eric Y. Chuang, Chia-Ti Tsai

**Affiliations:** 1 Department of Life Science, Genome and Systems Biology Degree Program, National Taiwan University, No. 1, Sec. 4, Roosevelt Rd., Taipei 106, Taiwan; cometrise@gmail.com; 2 Bioinformatics and Biostatistics Core, Center of Genomic Medicine, National Taiwan University, Taipei 100, Taiwan; 3 Department of Internal Medicine, National Taiwan University Hospital Yun-Lin Branch, Yun-Lin 640, Taiwan; b93401021@gmail.com (P.-S.H.); p95421008@ntu.edu.tw (S.-N.C.); 4 Division of Cardiology, Department of Internal Medicine, National Taiwan University College of Medicine and Hospital, No. 1, Section 1, Jen-Ai Road, Taipei 100, Taiwan; chokaiwu@ntuh.gov.tw (C.-K.W.); jueyhwang@ntu.edu.tw (J.-J.H.); 5 Graduate Institute of Clinical Medicine, College of Medicine, National Taiwan University, Taipei 100, Taiwan

**Keywords:** atrial fibrillation, thromboembolic stroke, copy number variation, genome-wide

## Abstract

Atrial fibrillation (AF) is a common cardiac arrhythmia and is one of the major causes of ischemic stroke. In addition to the clinical factors such as CHADS2 or CHADS2-VASC score, the impact of genetic factors on the risk of thromboembolic stroke in patients with AF has been largely unknown. Single-nucleotide polymorphisms in several genomic regions have been found to be associated with AF. However, these loci do not contribute to all the genetic risks of AF or AF related thromboembolic risks, suggesting that there are other genetic factors or variants not yet discovered. In the human genome, copy number variations (CNVs) could also contribute to disease susceptibility. In the present study, we sought to identify CNVs determining the AF-related thromboembolic risk. Using a genome-wide approach in 109 patients with AF and thromboembolic stroke and 14,666 controls from the Taiwanese general population (Taiwan Biobank), we first identified deletions in chromosomal regions 1p36.32-1p36.33, 5p15.33, 8q24.3 and 19p13.3 and amplifications in 14q11.2 that were significantly associated with AF-related stroke in the Taiwanese population. In these regions, 148 genes were involved, including several microRNAs and long non-recoding RNAs. Using a pathway analysis, we found deletions in *GNB1*, *PRKCZ*, and *GNG7* genes related to the alpha-adrenergic receptor signaling pathway that play a major role in determining the risk of an AF-related stroke. In conclusion, CNVs may be genetic predictors of a risk of a thromboembolic stroke for patients with AF, possibly pointing to an impaired alpha-adrenergic signaling pathway in the mechanism of AF-related thromboembolism.

## 1. Introduction

Atrial fibrillation (AF) is a common cardiac arrhythmia in the general population, and causes much morbidity and mortality [1]. Although patients with AF may have many different clinical symptoms, presentations, and complications, the most serious complication is a systemic thromboembolism, especially a thromboembolic stroke. Therefore, preventing AF-related thromboembolic events is an important issue. Many clinical factors have been found to be associated with the risk of thromboembolic stroke in patients with AF (e.g., CHADS2 or CHADS2-VASC score) [2]; however, these clinical risk factors cannot fully explain all the risks of stroke for patients with AF [3].

To date, the impact of genetic factors or polymorphisms on the risk of thromboembolic stroke in patients with AF has been largely unknown. Previously, we have shown that the angiotensinogen gene and CRP gene genetic polymorphisms could predict the risk of stroke for patients with AF [4,5]. In the human genome, the copy number variation (CNV) is a variation in the copy number of a specific chromosomal segment or DNA sequence and can affect the expression and function of genes in that chromosomal segment or its nearby segments [6], and is also associated with different phenotypes. It has been demonstrated that both single nucleotide polymorphisms (SNP)s and CNVs contribute to disease susceptibility [6]. Therefore, examining both SNP and CNV variants is important to determine the genetic causes of complex human phenotypes and diseases.

Around 5% of the human genome is covered by CNVs [7]. It has been shown that CNVs are also associated with common human diseases [8]. Whether CNVs may also contribute to the risk of thromboembolic stroke in patients with AF has not been addressed before. Accordingly, the objective of this study was to identify novel CNVs by a genome-wide approach that might be associated with the risk of thromboembolic stroke in patients with AF in the Taiwanese population.

## 2. Methods

### 2.1. Study Population and Outcome Assessments

The study population consisted of 16,000 subjects from the Taiwan Biobank (TWB) [9]. We excluded those subjects with cardiac diseases such as arrhythmia, cardiomyopathy, congestive heart failure or valvular diseases, and then there were 14,666 subjects left serving as controls. The baseline characteristics of these 14,666 subjects are summarized in Table 1. The mean age was 48 ± 11 years; among them, 7246 were male (49.4%). The TWB is a national database that is accessible to all the researchers in Taiwan to allow studies focusing on the relationships between genetics, environment, diet, and the causes and prognoses of diseases. The cohort was from the general Taiwanese population. The TWB database includes disease gene mapping, and therefore, genome-wide genotyping data are readily available and the number of recruited subjects continues to increase. TWB database is managed and regulated by the Ministry of Health and Welfare in Taiwan. All participants were of Han Chinese ancestry.

AF patients had been were enrolled since January 1998 at the National Taiwan University Hospital and the National Taiwan University Hospital Yun-Lin Branch (National Taiwan University Atrial Fibrillation Registry [NTUAFR]) [4,5,10]. The patients were followed up in the Cardiovascular Clinic through December 2013. The diagnosis of AF was documented by electrocardiograms (ECGs) and/or ambulatory ECG monitoring. Patients with symptoms of palpitations and that were suspected of AF were excluded if there was no ECG documentation of AF. The exclusion criteria also included receiving postmenopausal hormone replacement therapy, thyroid dysfunction, uncontrolled hypertension, cancer within the previous 5 years, rheumatoid arthritis, major surgery within the previous 1 month, and major infection with sepsis. The study protocols were reviewed and approved by the institutional review committee, and the study subjects agreed to participate in the study.

The endpoint or primary outcome during follow-up was defined as events of ischemic stroke. The definition of ischemic stroke was sudden-onset, focal or global neurologic deficits with supporting evidence from the brain imaging studies, such as the computed tomography or magnetic resonance image. Although the ischemic strokes include the large-artery atherosclerosis or small-vessel occlusions, both were included as the primary outcome. Hemorrhagic stroke was excluded.

In this study, among the patients developing adverse thromboembolic events, 109 patients with ischemic stroke received genome-wide SNP genotyping study and were selected as the case population. The clinical characteristics of these 109 patients are also summarized in Table 1. The mean age was 71 ± 12. Among them, 53 were males (48.6%). The cases and controls had similar clinical characteristics, except that the controls were younger than the cases.

Written informed consent was collected from each participating individual. This study was approved by the Institutional Review Board (IRB) on Biomedical Science Research/IRB-BM Academia Sinica, Taiwan, and National Taiwan University Hospital, and by the Ethics and Governance Council (EGC) of Taiwan Biobank, Taiwan.

### 2.2. DNA Extraction and Genome-Wide Detection of CNV

Genomic DNA was extracted by the standard non-enzymatic method [11]. Using the Axiom Genome-Wide TWB Array Plate (653,291 markers) SNP-based probes (Affymetrix, Santa Clara, CA, USA), genome-wide genotyping was performed. CNV regions were first identified through comparing the allelic intensities between the cases and controls. Affymetrix Power Tools (APT) version 2.10.0 (Affymetrix, Santa Clara, CA, USA) (https://www.affymetrix.com/support/developer/powertools/changelog/index.html) were used to process all CEL files generated by Axiom Genome-Wide TWB Array. APT was used for QC and transforming CEL files to summary files. First, we generated sample Dish QC (DQC) values. Next, samples with a DQC value less than the default DQC threshold were excluded. Next, in order to get the highest quality genotyping data, additional poor samples should be filtered out. The most basic post-genotyping filter was based on the sample QC call rate. Samples with a QC call rate value less than the default threshold of 97% were excluded. All the QC steps followed the Axiom™ Genotyping Solution DATA ANALYSIS GUIDE (Affymetrix, Santa Clara, CA, USA). We used multiple criteria from PARTEK Genomic Suite 6.6 (PARTEK, St. Louis, MO, USA), which is based on an algorithm of segmentation, to increase the sensitivity of CNV identification. We used our previously reported criteria to define the CNV segments [12,13]: (1) Regions with significantly different average intensities from those of nearby regions with the significant level of *p* value < 0.001; (2) breakpoints or region boundaries defined as those with the smallest *p* value; and (3) signal-to-noise ratio ≥ 0.3. SNPs with a smoothing value below and above 2 ± 0.3 were defined as loss and gain of copy number, respectively. The approximate CNV breakpoints were predicted empirically and were defined at the midpoint between the two adjacent boundaries of the two nearby segments. The size of the CNV was defined as the genomic length between the 5’ and 3’ breakpoints. Each CNV region in the genome was annotated according to NCBI RefSeq (hg19).

### 2.3. Pathway Analysis

While so many components are implicated in the mechanism of a complex trait disease, it is difficult to infer which components play a determining role. In this scenario, pathway analysis is a tool used to identify possible biological pathways involving related components. Ingenuity Pathway Analysis (IPA) is a pathway-search tool that identifies candidate related targets or biomarkers within the biological systems and has been designed for the analysis and interpretation of omics data. Therefore, in the present study, to derive a possible pathway that may be implicated in the mechanism of AF related thromboembolic stroke, data were analyzed through the use of IPA (QIAGEN, Germantown, MD, USA) [14]. The significance values for the canonical pathways in IPA was calculated by the Fisher’s exact test. The significance level indicates the probability of association of the identified genes with the canonical biological pathway by random chance alone. Therefore, the lower probability level indicates a higher significance of the identified pathway.

### 2.4. Statistical Analysis

All data were presented as percentages or mean ± standard deviations. We compared between-group categorical variables using the chi square test and continuous variables using the Student’s *t* test. The association of each CNV with the disease phenotype was investigated using the logistic regression analysis to adjust for the possible confounding effect of age and gender. The copy number of each CNV was incorporated into the regression model as a continuous covariate assuming a normal CN of 2. Because a total of 5304 CNVs were identified at the genome-wide level, a *p* value of < 0.05/5304 was considered statistically significant after Bonferroni’s correction for multiple test. The R statistical software was used for the statistical analyses.

## 3. Results

### 3.1. General CNV Pattern and Association with the Risk of Thromboembolic Stroke Induced by AF

A genome-wide detection of CNV was performed in 109 patients with AF and thromboembolic stroke and 14,666 controls, using the Axiom Genome-Wide TWB Array Plate (653,291 markers) to obtain SNP and allelic fluorescence intensities and to generate CNV calls and determine copy number. We removed markers or subjects with a call rate <95% for quality control filtering. We identified a total of 5304 CNVs. The CNV calls spanned between 1 and 2228 SNP markers, with an average CNV region size of 467 kb. Most of the identified CNVs were large CNVs (>500 kb).

**Figure 1:**
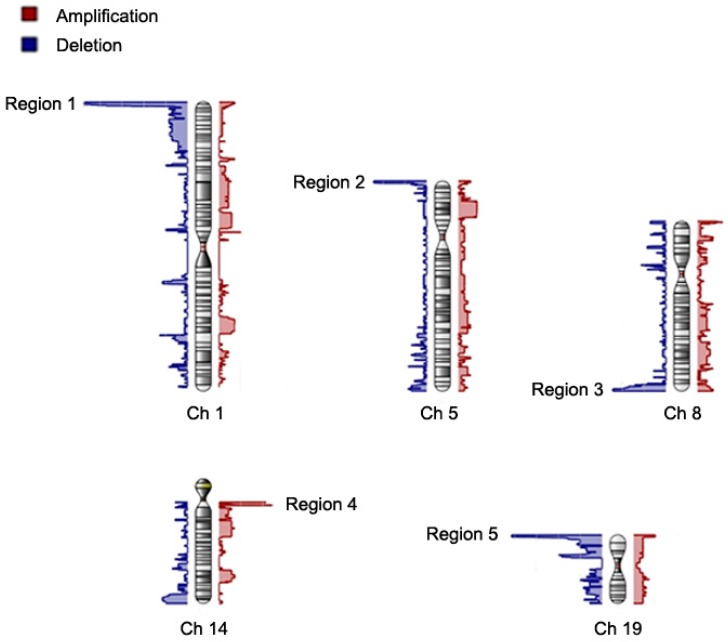
The chromosome view of genome-widely identified copy number variations including deletions and amplifications. The identified copy number variations are filtered using criteria of presence of total copy number aberration in more than 15% of the patients and minor allele frequency more than 0.15. After filtering, there are 5 significant regions (regions 1–5) in chromosome 1 (Ch 1), 5 (Ch 5), 8 (Ch 8), 14 (Ch 14) and 19 (Ch 19) in which the copy numbers are significantly different between atrial fibrillation (AF) patients with thromboembolic stroke and controls. X axis represents the number of aberration patients. Blue and red regions represent deletion and amplification, respectively.

We then focused on those CNVs with a total aberration present in more than 15% of the cases and minor allele frequency more than 0.15. The results are shown in Figure 1 and Table 2. Figure 1 shows the chromosomal views of all the identified CVNs. After filtering, there were 31 significant CNV segments left clustering in 5 chromosomal regions (5 peaks in chromosomal views), which affected 148 genes (Supplementary Table S1). Most of these CNV segments localized at the ends of chromosomes. Of these 31 CNV segments, 28 CNV segments were deletion and 3 amplification. The average copy number was 1.38 (deletion) and 2.46 (amplification), respectively (Table 2). Most CNV segments are found in 1p36.32, 1p36.33, and 19p13.3.

### 3.2. Functional Integration of Identified CNVs and Genes by Pathway Analysis

**Figure 2:**
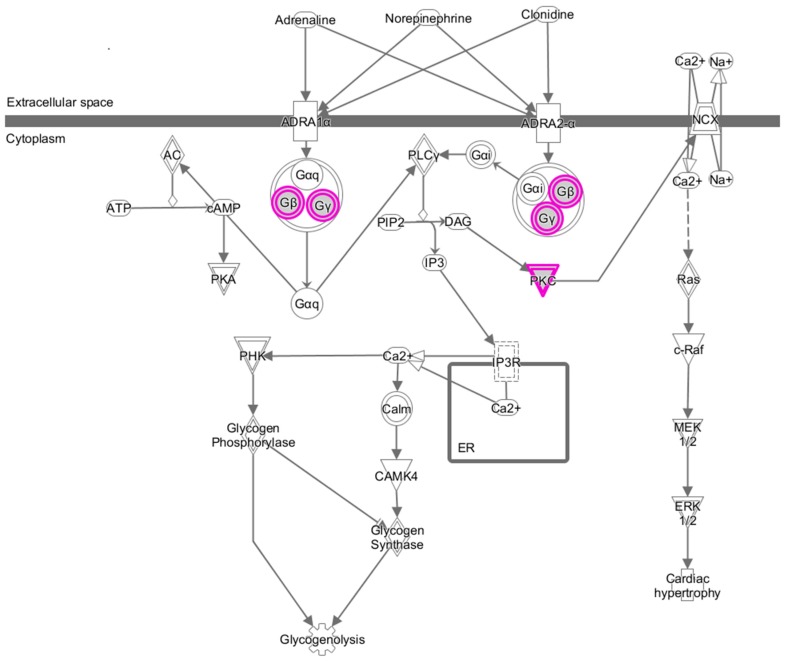
An illustration depicting detailed alpha-adrenergic receptor (ADR) signaling pathways. Purple circles represent the genes in which one allele deletion is more common in patients with atrial fibrillation related thromboembolic stroke compared to controls. *AC*, 3′,5′-cyclic *AMP* synthetase; *ADRA1α*, adrenergic Receptor alpha1a; *ADRA2-α*, adrenergic receptor, alpha2a; *ATP*, adenosine 5′-triphosphate; *c-Raf*, serine/threonine kinase; *CAMK4*, calcium/calmodulin-dependent protein kinase IV; *cAMP*, cyclic-3′,5′-monophosphate; *DAG*, diacylglycerides; *ERK1/2*, p42/p44 MAP kinase; *Gαi*, G protein alpha I subunits; *Gαq*, alpha q polypeptide; *Gαq-Gβ-Gγ*, G protein alpha beta gamma; *Gβ*, G-protein beta subunits; *Gγ*, G protein gamma subunits; *IP3*, inositol 1,4,5-triphosphate; *IP3R*, Inositol 1,4,5-triphosphate receptor; *NCX*, Na+/Ca+ exchanger; *PHK*, Glycogen phosphorylase kinase; *PIP2*, 1-phosphatidyl-1D-myo-inositol 4,5-bisphosphate; *PKA*, cAMP-dependent protein kinase; *PKC*, protein kinase C; *PLCγ*, Phospholipase C gamma.

The functions of all the identified 148 genes are shown in Supplementary Table S1. The functions of involved genes were very diverse, including microRNAs and long non-coding RNAs. Because there were so many affected genes that might be implicated in the mechanism of AF related thromboembolic stroke, we further did a pathway analysis to elucidate the possible translational meaning and significance. The result of in silico pathway analysis is shown in Figure 2.

Interestingly, we found perturbation of alpha-adrenergic receptor (ADR) signaling pathway (*p*-value = 9.7 × 10^−3^) in patients with AF and thromboembolic stroke. Genes involved in this pathway were G protein subunit beta 1 (*GNB1*), protein kinase C zeta (*PRKCZ*) and G protein subunit gamma 7 (*GNG7*) (Table 3). These CNVs are common diallelic CNVs and have also been published in the Database of Genomic Variants (http://projects.tcag.ca/variation). In this study, most of the AF patients with thromboembolic stroke had deletion alleles in these 3 genes. Therefore, concomitant deletion in these 3 genes might be associated with an increased risk of AF-related thromboembolic stroke.

## 4. Discussion

The most serious complication of AF is the thromboembolic stroke, which occurs with increasing frequency when risk factors, either clinical or genetic risk factors, accumulate. Currently, the major treatment for AF related thromboembolic stroke is oral anti-coagulant, which could not reduce the risk to that of those without AF and is also associated with serious bleeding complications. When we better know the detailed molecular mechanism of AF-related thromboembolic stroke, we may have an opportunity to find a better treatment strategy for this serious complication.

In the present study, we first demonstrated the possible role of ADR in the molecular mechanism of AF related thromboembolic stroke. More deletions of genes encoding proteins of ADR signaling were found in patients with AF related thromboembolic stroke. Therefore, we hypothesized that the impairment of ADR signaling and function may predispose AF patients to develop a thromboembolic stroke. However, how does impaired ADR signaling contribute to the susceptibility to thromboembolic stroke in patients with AF?

ADRs are divided into several distinct pathways, such as ADR-α and ADR-β [15]. ADR-α is coupled through G-proteins and activation of protein kinase C (PKC) whereas ADR-β is coupled to activation of adenyl cyclase and protein kinase A (PKA). ADR-α is involved in many physiological functions including the cardiovascular system through a calcium dependent mechanism. ADR-α activates calmodulin and calmodulin dependent protein kinase (CaMK) through PKC and calcium, resulting in the activation of many calcium dependent molecular effects.

AF is a complex arrhythmia and there must be factors to facilitate the maintenance and self-perpetuation of this complex arrhythmia, which have been known as atrial remodeling [16]. After a prolonged duration of AF, the atria undergo changes in structural, electrophysiological and mechanical properties, which contribute to the maintenance of AF and are called structural, electrical and contractile remodelings, respectively [16]. Among them, contractile remodeling results in impaired atrial contractile function and stasis of blood in the atria, which consequently promotes thrombus formation and systemic embolization [17,18].

In animal models with sustained AF, there was a correlation between the decrease of contraction or shortening of atrial myocytes and the reduction of the amplitude of the calcium transient [19]. The L-type calcium current (ICaL) was also down regulated significantly [20]. Since the ICaL is a main factor in determining the amplitude of calcium transient or calcium amount released from the sarcoplasmic reticulum (SR), the down-regulation of ICaL may contribute to the contractile dysfunction or remodeling during AF.

It has been demonstrated that calmodulin and CaMK play an important role in regulating ICaL during AF [21,22]. Hence, decreased calmodulin and CaMK signaling has been implicated in the mechanism of atrial remodeling in AF [23]. Therefore, it is logical to speculate that the deletion of ADR-α related genes may result in impairment of ADR-α signaling, decreased calmodulin and CaMK signaling and reduced ICaL, which consequently contributes to atrial contractile dysfunction and thrombus formation and predisposes the AF patients to develop a thromboembolic stroke.

In the present study, all the identified significant CNVs localized to the ends of chromosomes (Figure 1). It has been shown that CNVs at the ends of chromosomes (terminal deletion) are associated with certain human diseases, such as structural brain abnormalities, cardiovascular disease, and psychomotor retardation [24,25,26,27,28]. At present, there are no reports addressing that CNVs are more common or preferentially localize at the ends of chromosomes. Therefore, the localization of the identified significant CNVs at the ends of chromosomes in the present study is not a non-specific effect. We speculate that CNVs at the ends of chromosomes are associated with AF related thromboembolic stroke, as the terminal 4q deletion syndrome [24,25,26]. This finding should be further validated in other studies.

There are limitations in the present study. First, the case number of patients with AF and thromboembolic stroke is low in our study. In our previous study [29], we demonstrated that significant disease-associating CNVs could be identified in samples with a case number as low as 100 patients. Nevertheless, our results should be replicated and validated in larger populations and in other ethnic populations. Second, the mechanisms by which the identified genes are linked to AF-related thromboembolic stroke are largely unknown. Future molecular studies, especially focusing on ADR-α signaling, calmodulin dependent protein kinase, atrial contractile dysfunction and thrombus formation are warranted in the future.

## Figures and Tables

**Table 1:** Clinical characteristics.

Variables	AF with Stroke (*n* = 109)	Controls (*n* = 14,666)
Age (yr) *	71 ± 12	48 ± 11
Male (%)	53 (48.6)	7246 (49.4)
DM (%)	9 (8.3)	656 (4.4)
HTN (%)	18 (16.5)	1589 (10.8)
HPL (%)	11 (10.1)	848 (5.7)

AF, atrial fibrillation; DM, diabetes mellitus; HPL, hyperlipidemia; HTN, hypertension; yr, years; * *p* < 0.05 for comparison between AF subjects and controls.

**Table 2:** Significant CNV regions in the 109 AF samples.

CNV Region	Cytoband	Start Position	Type	Allele Frequency	Avg CN	Length (bps)	Nearest Feature	*p*	ln(OR) (95% CI)
Region 1	1p36.33	565433	Del	0.30	1.45	1,614,263	*GNB1*, *PRKCZ*	1.69 × 10^−10^	15.3 (12.9–17.7)
	1p36.33-1p36.32	2179695	Del	0.30	1.45	298,359	*RP3-395M20.12*	2.14 × 10^−11^	17.5 (14.9–20.1)
	1p36.32	2478053	Del	0.28	1.44	391,244	*RP11-740P5.3*	2.95 × 10^−9^	15.8 (13.1–18.4)
	1p36.32	2869296	Del	0.27	1.43	293,652	*PRDM16*	6.24 × 10^−9-^	13.8 (11.5–16.2)
	1p36.32	3162947	Del	0.26	1.42	10,249	*PRDM16*	8.58 × 10^−9^	13.8 (11.4–16.2)
	1p36.32	3173195	Del	0.24	1.41	184,908	*PRDM16*	1.44 × 10^−8^	13.8 (11.3–16.2)
	1p36.32	3358102	Del	0.24	1.41	297,333	*TP73-AS1*	5.09 × 10^−9^	15.8 (13.1–18.5)
	1p36.32	3655434	Del	0.23	1.41	7371	*TP73-AS1*	5.53 × 10^−9^	15.8 (13.1–18.5)
	1p36.32	3662804	Del	0.22	1.41	18,596	*CCDC27*	3.44 × 10^−7^	14.4 (11.6–17.2)
	1p36.32	3681399	Del	0.21	1.40	11,306	*SMIM1*	3.86 × 10^−7^	14.4 (11.6–17.3)
	1p36.32	3692704	Del	0.20	1.39	3565	*SMIM1*	5.03 × 10^−7^	14.4 (11.5–17.2)
	1p36.32	3696268	Del	0.19	1.38	45,605	*CEP104*	5.74 × 10^−7^	14.4 (11.5–17.3)
	1p36.32	3741872	Del	0.17	1.36	58,040	*DFFB*	1.2 × 10^−5^	11.9 (9.2–14.7)
	1p36.32	3799911	Del	0.17	1.36	205,245	*RP13-614K11.1*	1.53 × 10^−5^	11.9 (9.2–14.7)
Region 2	5p15.33	1313855	Del	0.16	1.39	158,260	*LPCAT1*	1.9 × 10^−5^	10.0 (7.7–12.4)
	5p15.33	1472114	Del	0.16	1.39	193,377	*RP11-43F13.1*	5.24 × 10^−5^	13.3 (10.0–16.6)
Region 3	8q24.3	144283121	Del	0.16	1.43	357,744	*GSDMD*	5.56 × 10^−6^	8.5 (6.6–10.4)
Region 4	14q11.2	22754700	Amp	0.15	2.45	1721	*TRAV38-2DV8*	8.23 × 10^−7^	12.6 (10.0–15.1)
	14q11.2	22756420	Amp	0.16	2.46	178,718	*TRDC*	1.79 × 10^−9^	14.6 (12.1–17.0)
	14q11.2	22935137	Amp	0.15	2.46	7248	*TRDV3*	1.68 × 10^−8^	14.2 (11.7–16.7)
Region 5	19p13.3	2089647	Del	0.20	1.30	38,105	*AP3D1*	1.64 × 10^−6^	6.9 (5.5–8.3)
	19p13.3	2127751	Del	0.20	1.34	23,119	*AP3D1*	6.25 × 10^−7^	8.2 (6.5–9.8)
	19p13.3	2150869	Del	0.19	1.35	19,047	*DOT1L*	3.97 × 10^−8^	11 (9.0–13.0)
	19p13.3	2169915	Del	0.19	1.35	945	*DOT1L*	3.33 × 10^−7^	7.4 (6.0–8.9)
	19p13.3	2170859	Del	0.19	1.35	139,329	*LINGO3*	2.2 × 10^−7^	8.4 (6.8–10.5)
	19p13.3	2310187	Del	0.19	1.36	4407	*LSM7*	3.57 × 10^−8^	11.0 (9.0–13.0)
	19p13.3	2314593	Del	0.17	1.35	86,339	*TMPRSS9*	1.44 × 10^−6^	9.9 (7.8–11.9)
	19p13.3	2400931	Del	0.17	1.33	3631	*TMPRSS9*	2.7 × 10^−6^	9.7 (7.7–11.8)
	19p13.3	2404561	Del	0.16	1.32	73,270	*GADD45B*	6.5 × 10^−6^	9.5 (7.4–11.7)
	19p13.3	2477830	Del	0.15	1.32	30,337	*RNU6-993P*	8.82 × 10^−6^	9.5 (7.4–11.7)
	19p13.3	2508166	Del	0.15	1.34	21,100	*GNG7*	3.21 × 10^−6^	10 (7.8–12.1)

AF, Atrial fibrillation; Amp, amplification; Avg CN, averaged copy number in cases; bps, base pairs; CNV, copy number variation; Del, deletion; ln(OR) (95% CI), logarithmic transformation of odds ratio and its 95% confidence interval.

**Table 3:** Three genes involved in ADR signaling pathway.

Gene Symbol	Cytoband	Entrez Gene Name	Location	Type(s)
*GNB1*	1p36.32	G protein subunit beta 1	Plasma Membrane	enzyme
*GNG7*	19p13.3	G protein subunit gamma 7	Plasma Membrane	enzyme
*PRKCZ*	1p36.32	protein kinase C zeta	Cytoplasm	kinase

ADR, alpha-adrenergic receptor.

## Supplementary Materials

The following are available online at https://www.mdpi.com/2077-0383/8/3/332/s1, Table S1: Detail information of significant CNV segments.
